# Altered Regulation of Akt Signaling with Murine Cerebral Malaria, Effects on Long-Term Neuro-Cognitive Function, Restoration with Lithium Treatment

**DOI:** 10.1371/journal.pone.0044117

**Published:** 2012-10-17

**Authors:** Minxian Dai, Brandi Freeman, Henry J. Shikani, Fernando Pereira Bruno, J. Elias Collado, Rolando Macias, Sandra E. Reznik, Peter Davies, David Conover Spray, Herbert Bernard Tanowitz, Louis Martin Weiss, Mahalia Sabrina Desruisseaux

**Affiliations:** 1 Department of Pharmaceutical Sciences, College of Pharmacy and Allied Health Professions, St John's University, Queens, New York, United States of America; 2 Department of Pathology, Albert Einstein College of Medicine, Bronx, New York, United States of America; 3 Pontificia Universidad Catolica Madrey Maestra, Santiago, Dominican Republic; 4 Meharry Medical College, Nashville, Tennessee, United States of America; 5 The Dominick P. Purpura Department of Neuroscience, Albert Einstein College of Medicine, Bronx, New York, United States of America; 6 Litwin-Zucker Center for the Study of Alzheimer's Disease and Memory Disorders, Feinstein Institute for Medical Research, Manhasset, New York, United States of America; 7 Department of Medicine, Albert Einstein College of Medicine, Bronx, New York, United States of America; University of Oklahoma Health Sciences Center, United States of America

## Abstract

Neurological and cognitive impairment persist in more than 20% of cerebral malaria (CM) patients long after successful anti-parasitic treatment. We recently reported that long term memory and motor coordination deficits are also present in our experimental cerebral malaria model (ECM). We also documented, in a murine model, a lack of obvious pathology or inflammation after parasite elimination, suggesting that the long-term negative neurological outcomes result from potentially reversible biochemical and physiological changes in brains of ECM mice, subsequent to acute ischemic and inflammatory processes. Here, we demonstrate for the first time that acute ECM results in significantly reduced activation of protein kinase B (PKB or Akt) leading to decreased Akt phosphorylation and inhibition of the glycogen kinase synthase (GSK3β) in the brains of mice infected with *Plasmodium berghei* ANKA (PbA) compared to uninfected controls and to mice infected with the non-neurotrophic *P. berghei* NK65 (PbN). Though Akt activation improved to control levels after chloroquine treatment in PbA-infected mice, the addition of lithium chloride, a compound which inhibits GSK3β activity and stimulates Akt activation, induced a modest, but significant activation of Akt in the brains of infected mice when compared to uninfected controls treated with chloroquine with and without lithium. In addition, lithium significantly reversed the long-term spatial and visual memory impairment as well as the motor coordination deficits which persisted after successful anti-parasitic treatment. GSK3β inhibition was significantly increased after chloroquine treatment, both in lithium and non-lithium treated PbA-infected mice. These data indicate that acute ECM is associated with abnormalities in cell survival pathways that result in neuronal damage. Regulation of Akt/GSK3β with lithium reduces neuronal degeneration and may have neuroprotective effects in ECM. Aberrant regulation of Akt/GSK3β signaling likely underlies long-term neurological sequelae observed in ECM and may yield adjunctive therapeutic targets for the management of CM.

## Introduction

Cerebral malaria (CM) resulting from infection with *Plasmodium falciparum* remains one of the deadliest diseases in the developing world, resulting in nearly 1 million annual deaths worldwide. In addition, CM has become a significant cause of long-term neuro-cognitive deficits in survivors, despite successful eradication of the parasite [1]–[10]. The mechanisms that underlie the lingering effects of CM after successful anti-parasitic treatment remain largely unknown. Vasculopathy with subsequent ischemia has been proposed as a possible etiology [11]–[14]. Recent microarray analysis suggests that neuronal and glial disturbances may also be etiologic in the development of ECM [15]. A little-recognized effect of CM is the metabolic dysfunction that occurs as a result of this vasculopathy and the neuronal damage which ensues. Primate studies of CM have demonstrated metabolic abnormalities in brains of infected animals with impairment in glucose uptake preceding parenchymal damage or manifestations of ECM [16]. Our previous studies in a murine model of CM demonstrating that n-acetyl aspartate (NAA), an inverse indicator of both of neuronal loss and recent or ongoing neuronal injury/dysfunction [17]–[19], is decreased in the brains of mice with CM reflects this impairment of metabolic function in affected neurons [20].

Certain neuronal proteins and markers of impaired metabolism have been implicated in CM as indicators of disease severity. Medana et al demonstrated that increased levels of the microtubule (MT)-associated protein tau correlate with pronounced cerebral pathology and coma as well as with adverse systemic organ involvement in both children and adults with CM [21], [22]. Tau is a key protein in the formation of intra-neuronal and glial fibrillary lesions that are the hallmark of Alzheimer's disease and other neurodegenerative diseases. [23]–[27]. The regulation of tau is very important, as its dysregulation has been linked to cerebral inflammation and ischemia, as well as insulin resistance [28]–[31].

Tau regulation is modulated by many kinases, of which the glycogen synthase kinase (GSK3β) is likely the most important. GSK3β is ubiquitously active and is a critical effector of PI3K/Akt cellular signaling. Several cellular processes such as cell metabolism, cell death and survival depend on GSK3β. It has been implicated in diabetes as well as neurodegenerative diseases including Alzheimer's disease [32]–[37]. Upon phosphorylation at Ser9 by Akt, also called protein kinase B, GSK3β becomes inactive [32]. Akt is a serine/threonine kinase that is an important regulator of cell survival and apoptosis. It is also a critical effector of insulin and growth factor mediated neuronal survival [38] as it is vital in insulin signaling and is required for insulin-induced translocation of glucose transporter 4 (GLUT4) to the plasma membrane [39], [40]. Akt has been shown to be a critical regulator of parasite development and survival in the mosquito as activation of the enzyme in the mosquito midgut not only significantly reduces *P. falciparum* load, but also reduces mosquito lifespan [41].

The notion that intracellular parasites are capable of regulating cellular function by affecting host metabolism pathways is certainly not new. For example, Wang et al demonstrated that the intracellular protozoan *Toxoplasma gondii* induces host cell growth via alteration of mTOR signaling [42], and *Trypanosoma cruzi* has been noted to activate the PI3K/Akt pathway in adipocytes [43]. However, malaria parasites have never previously been reported to affect mammalian host survival pathways. We demonstrate for the first time, in a murine model, that although malaria parasites do not invade tissue, experimental cerebral malaria (ECM) triggers alterations in the Akt/GSK3β cell survival/insulin signaling pathway, and that this induces profound changes in the regulation of neuronal function and survival. We propose that this mechanism underlies the long-term neurological sequelae that persist in survivors of CM. The Akt/GSK3β survival pathway presents potential therapeutic targets for pharmaceutical agents which might be used as adjunctive therapy to bring about salvage of neurological function in children with CM.

## Results

### Parasitemia and mortality

At the administered dose, lithium did not confer any anti-parasitic effect, and the parasite response to chloroquine (CQ) therapy was similar in both saline (NaCl) and lithium (LiCl) treated infected mice (See supplemental material; Fig. S1A). In addition, survival was similar in both NaCl and LiCl treated mice (supplemental material, Fig. S1B).

### Histological analysis

**Figure 1:**
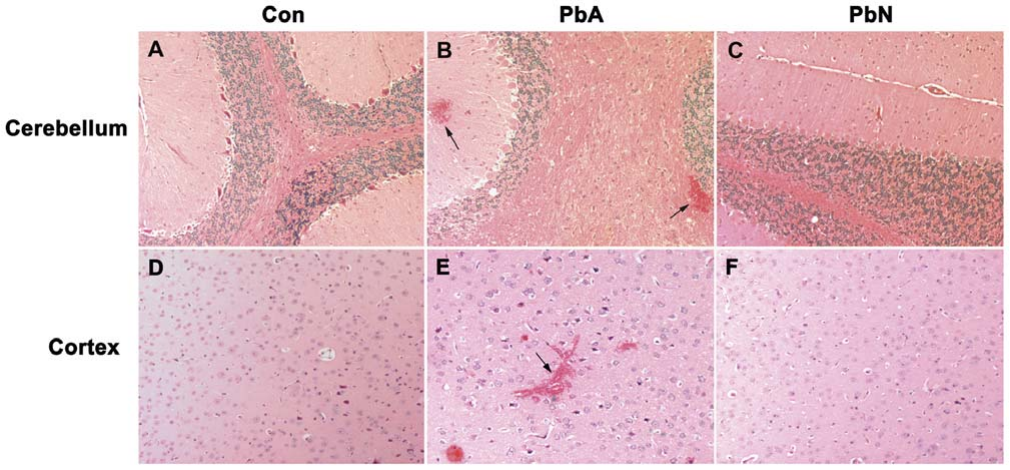
Pathology observed during ECM. **A–C:** While control mice and PbN-infected mice demonstrated no hemorrhage in the brain, PbA infection resulted in significant degrees of hemorrhage in the cerebellum (F(_2, 11)_ = 7.84; p<0.01) **(B- arrows)**. **D–F:** PbA infection also resulted in significantly higher hemorrhage **(E- arrow)** in the areas associated with cognitive function, e.g. cerebral cortex **(D–F)**, corpus callosum, hippocampus, fornix and thalamus (F_(2, 12)_ = 5.29; p<0.05). Semi-quantitative determination of hemorrhage as previously published [44]. n = 4 Con, 7 PbA, 3 PbN. Con = control, PbA = *P. berghei* ANKA infected mice, PbN = *P. berghei* NK65 infected mice.

Consistent with our previous publications [43], [44], hematoxylin-eosin (H&E) staining of the coronal sections of PbA-infected mice demonstrated significant hemorrhage in several regions of the brain including cerebellum (Fig. 1A–C), the brainstem as well as in regions of the brain associated with cognitive dysfunction. Though individually, the regions most commonly associated with cognitive function, such as the cortex, corpus callosum, hippocampus, fornix and thalamus, demonstrated no increase in hemorrhage in PbA-infected mice, PbA infection conferred a significantly higher degree of hemorrhage compared to control or to PbN infection when using combined data from those regions (Fig. 1D–F). This was consistent with our prior observations [44].

### Alterations in Akt/GSK3β signaling with acute ECM

Glycogen synthase kinase 3 (GSK3β) is a ubiquitously active enzyme which is inhibited upon phosphorylation at Ser9 by activated protein kinase B (PKB or Akt). Akt is a crucial regulator of cellular proliferation and survival as well an important factor in insulin signaling. In order to determine the effect of malarial infection on cell survival regulatory proteins, we examined the kinases involved in the Akt pathway in the brains of mice at day eight post-infection (dpi) with *Plasmodium berghei* ANKA (PbA) in comparison to that of mice infected with the non-neurotrophic *P. berghei* NK65 (PbN) and in uninfected control mice.

**Figure 2:**
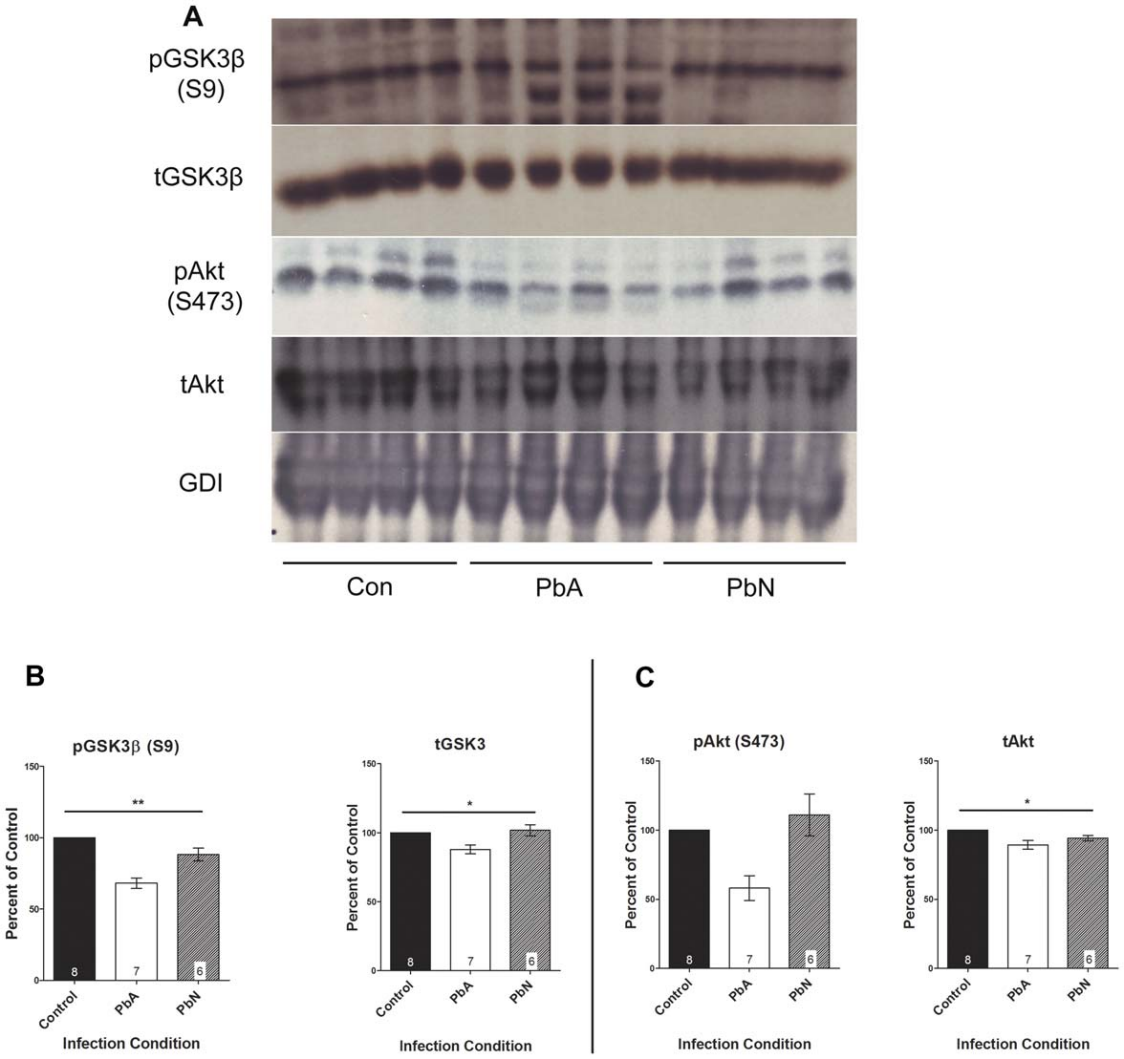
Representative immunoblots of GSK3β and Akt expression. (**A**) Brain lysates were probed with antibodies to phospho-GSK3β at Ser9 (pGSK3β (S9)), total GSK3β, phospho-Akt at Ser473 (pAkt (S473)), and total Akt. (**B**) Densitometry measurements of immunoblots demonstrated that PbA infection resulted in significantly lower expression of pGSK3β (S9) (F_(2, 18)_ = 19.87; p<0.001) when compared to uninfected control mice or to mice infected with PbN. PbA-infected mice demonstrated a 32% decrease in phospho-GSK3β (S9) expression compared to controls (n = 8 Con, 7 PbA) and a 23% decrease compared to mice infected with PbN (n = 7 PbA, 6 PbN). This was associated with general effects of infection condition on the mean protein expression of total GSK3β (F_(2, 18)_ = 3.63; p<0.05); however, post-hoc Tukey's multiple comparison did not demonstrate any specific effects of individual conditions when comparing the different treatment groups. (**C**) There was a corresponding 42% decrease in the expression of pAkt (S473) in PbA-infected mice compared to control, and a 48% decrease compared to PbN-infected mice, though this did not reach statistical significance (F_(2, 17)_ = 2.79; p = 0.09). Interestingly, there were significant effects of PbA infection on the expression of total Akt (F_(2, 18)_ = 5.84; p<0.05), with Tukey's post-hoc analysis demonstrating significant reduction due to PbA when compared to uninfected controls. No differences were observed between PbA and PbN infected mice or between control and PbN infected mice. Total Akt and GSK3β protein expression levels were normalized to GDI, and absolute total protein levels were used to normalize respective phosphorylated protein levels. All densitometry measurements are illustrated as a percentage of corresponding measurements in uninfected controls. Values plotted as mean ± standard error (SEM). *p<0.05, **p<0.001. Con = control, PbA = *P. berghei* ANKA infected mice, PbN = *P. berghei* NK65 infected mice. GDI was used as loading control.

Densitometry measurements of immunoblots demonstrated that PbA infection resulted in significantly lower expression of phosphorylated GSK3β at Ser9 (pGSK3β (S9); Fig. 2A–B; p<0.001) when compared to uninfected control mice or to mice infected with PbN. PbA-infected mice demonstrated a 32% decrease in phospho-GSK3β (S9) expression compared to controls and a 23% decrease compared to mice infected with PbN. There was no effect of PbN infection on the phosphorylation of GSK3β at Ser9, illustrating that ECM results in decreased inhibition of GSK3β (Fig. 2B). This was associated with general group effects on the mean protein expression of total GSK3β (p<0.05), although there were no specific effects of infection condition when comparing the different treatment groups by Tukey's multiple comparison test (Fig. 2A–B).

In addition, PbA infection resulted in 42% lower expression of phosphorylated Akt at Ser473 Akt in whole brain lysates when compared to controls and 48% lower levels compared to PbN infected mice though this was not statistically significant (Fig. 2C; p<NS). Interestingly, however, There was a modest but significant effect of PbA infection on the expression of total Akt with PbA-infected mice exhibiting 11% less total protein expression than uninfected control mice (Fig. 2C; p<0.05).

**Figure 3:**
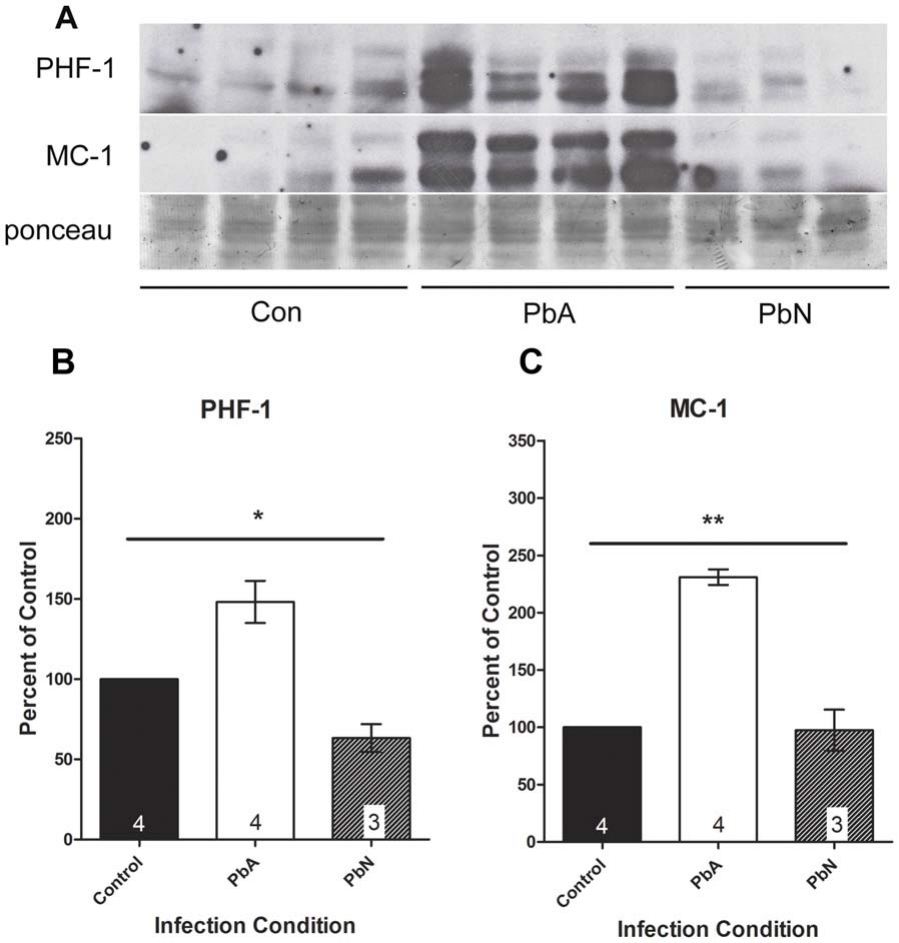
Abnormal tau expression in ECM. Aberrantly phosphorylated tau was evident in PbA infected mice when compared to control and PbN infected mice. (**A**) Brain lysates were probed with antibodies to PHF-1 which stains for phosphorylated tau at Ser396/404, and MC-1 which recognized misfolded tau protein. (**B**) PbA infected mice demonstrated a 48% higher in PHF-1 immunoreactivity compared to controls and a 134% more compared to PbN. Significant group effects on the means was demonstrated by one-way ANOVA (F_(2, 8)_ = 14.85; p<0.01) with significant mean differences between PbA mice and both control and PbN mice using post-hoc Tukey's multiple comparison test. Tukey's test did not demonstrate a significant effect of PbN infection on the mean PHF-1 expression when compared to control mice. (**C**) PbA-infected mice displayed a 131% more MC-1 expression compared to controls and 137% higher expression compared to PbN-infected mice. One way ANOVA demonstrated significant group effects in the expression of MC-1 (F_(2, 8)_ = 51.29; p<0.001) with post-hoc Tukey's test demonstrating significant effect of PbA infection on MC-1 expression when compared to either control or PbN mice, but no effect of PbN infection when compared to controls. Densitometry measurements are illustrated as a percentage of corresponding measurements in uninfected controls. Values plotted as mean ± SEM. *p<0.05, **p<0.001. Con = control, PbA = *P. berghei* ANKA infected mice, PbN = *P. berghei* NK65 infected mice. Ponceau was used as loading control.

We previously demonstrated that ECM results in cognitive impairment associated with white matter damage in mice [43], [45]. The protein tau, a substrate of GSK3β, interacts with microtubules and is associated with early memory loss and cognitive dysfunction in neurodegenerative diseases. In order to further assess damage to neurons as a result of ECM, we examined expression and post-translational modifications of tau in whole brain lysates. Immunoblot analysis of the brains of mice infected with PbA demonstrated a distinct shift to phosphorylated tau protein (mAb PHF-1) as well as a conformational change in tau (MC-1) which was not evident in control or PbN infected mice (Fig. 3A). Significant group effects on the mean PHF-1 expression was demonstrated by one-way ANOVA (Fig. 3B; p<0.01) with significant differences between PbA mice and both control and PbN mice. Interestingly, Tukey's test did not demonstrate a significant effect of PbN infection on the mean PHF-1 expression when compared to control mice.

Correspondingly, MC-1 recognizes a unique conformational epitope of misfolded tau in which the N-terminus interacts with the tubulin binding domains at the C-terminus. One way ANOVA demonstrated significant group effects in the expression of MC-1 (Fig. 3C; p<0.001) with post-hoc Tukey's test demonstrating significant effect of PbA infection on MC-1 expression when compared to either control or PbN mice, but no effect of PbN infection when compared to controls, illustrating that the post-translational modifications of tau protein were specific to ECM, and not simply due to severe malaria disease.

**Figure 4:**
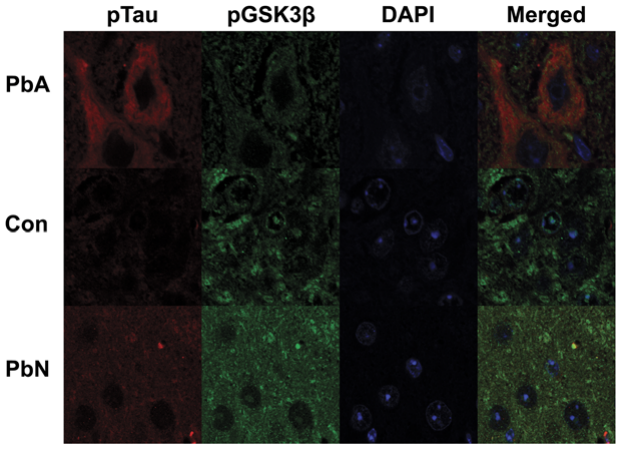
Immunofluorescence staining for phospho-tau (Red) and phospho-GSK3β (S9) (Green) in the brainstem. PbA-infected mice displayed an increase in tau-positive neurons when compared to control mice or mice infected with PbN and an overall decrease or absence of intra-nuclear staining of pGSK3β (S9) in those neurons.

Immunohistochemical staining of sagittal brain sections double-labeled with PHF-1 (which stains phosphorylated tau protein) and phospho-GSK3 in the region of the brainstem reveals that the decrease in phospho-GSK3β is at least partly attributable to a decrease in intranuclear expression of phospho-GSK3β in tau-positive neurons in PbA mice. PbA-infected mice demonstrated an absence of phospho-GSK3β staining in the nuclei of tau positive neurons whereas intranuclear phospho-GSK3β immunoreactivity was evident in control and PbN infected mice (Fig. 4), indicating that the aberrant phosphorylation and subsequent misfolding of tau is most likely a result of abnormal Akt regulation of GSK3β in the neurons.

These findings demonstrate that ECM induces a decrease in the protein expression of Akt in PbA infected mice with ensuing decrease of Akt phosphorylation and inhibition of GSK3β. This suggests that there is an alteration in the cell survival/insulin signaling pathway during ECM which results in post-translational modification of tau protein and neuronal degeneration.

### Lithium effects on long-term Akt/GSK3β signaling

We previously demonstrated a lack of inflammation and vascular damage in the brains of mice with persistent neuro-cognitive impairment, suggesting that other physiological factors may be etiologic in the development of cognitive deficits with ECM [44]. We examined the brains of mice after treatment with chloroquine (CQ) to assess the expression of Akt, GSK3β and tau. In addition we investigated the effect of lithium chloride (LiCl) on those proteins in uninfected and PbA-infected mice (ECM mice) treated with CQ. Lithium is a mood-stabilizing agent which induces phosphorylation and activity of Akt [46] in addition to its inhibitory effects on GSK3β [47]. As there were no differences in the mean densitometry intensities between CQ-treated uninfected mice treated with either LiCl or with sodium chloride (NaCl), the two control groups were combined.

**Figure 5:**
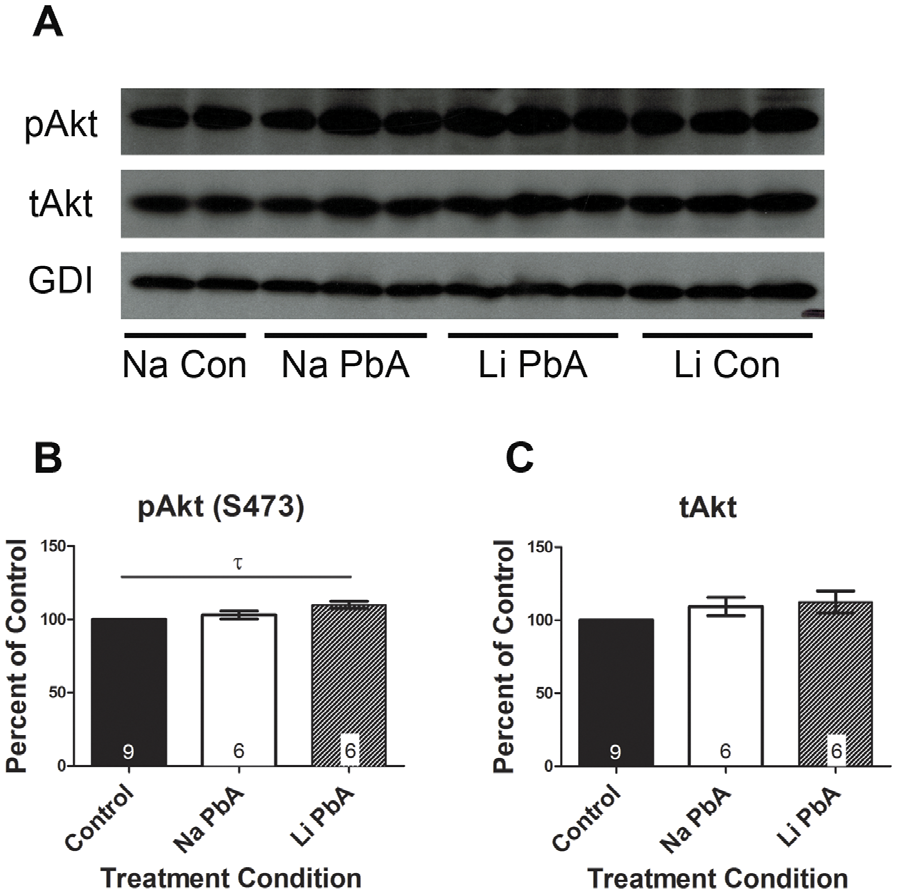
Expression of pAkt (S473) after chloroquine and lithium treatment. (**A–B**) expression of Akt phosphorylated at Ser473 reached control levels in ECM mice treated with NaCl. Tukey's analysis demonstrated a modest but significant increase in the expression of pAkt (S473) due to lithium treatment of ECM mice when compared to control (p<0.05), although a one-way ANOVA did not show significant overall group effects on the mean protein expression (F_(2, 18)_ = 3.361; p = 0.058). (**A, C**) There were no differences in total Akt expression between the groups. For the purposes of analysis, NaCl and LiCl treated control groups were combined as there were no significant differences in the means and no effect of treatment on mean protein expression. Total Akt protein expression levels were normalized to GDI. Absolute total Akt total protein levels were used to normalize respective pAkt (S473) levels. All densitometry measurements are illustrated as a percentage of corresponding measurements in uninfected controls. Values plotted as mean ± SEM. n = 4 Na Con, 6 Na PbA, 6 Li PbA, 5 Li Con. τ = significant Tukey's multiple comparison analysis (p<0.05). Na Con = NaCl treated control, Na PbA = NaCl treated *P. berghei* ANKA infected mice, Li PbA = LiCl treated *P. berghei* ANKA infected mice, Li Con = LiCl treated control. GDI was used as loading control.

Although the expression of Akt phosphorylated at Ser473 reached control levels in ECM mice treated with NaCl, Tukey's analysis demonstrated a modest but significant increase in the expression of phospho-Akt (S473) due to lithium treatment of ECM mice when compared to control (Fig. 5; p<0.05). Though one-way ANOVA did not reach statistical significance (p<0.058). There were no differences in total Akt expression between the groups (Fig. 5).

**Figure 6:**
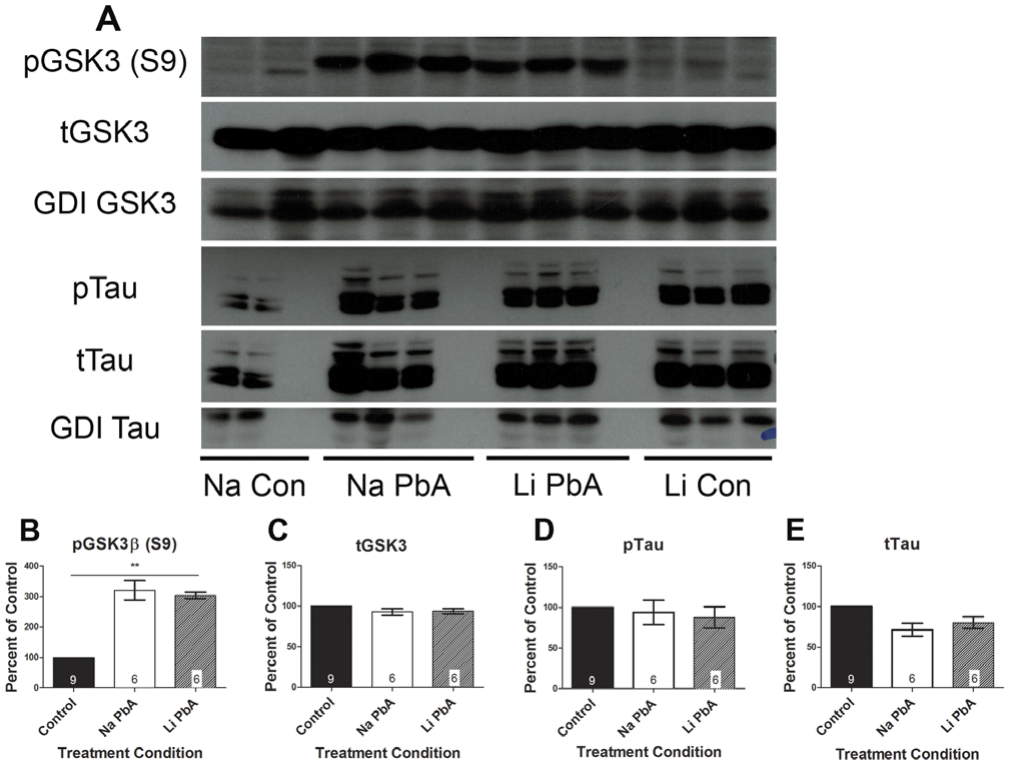
Expression of phospho-GSK3β and tau after chloroquine and lithium treatment. (**A–B**) PbA infected mice treated with NaCl demonstrated 220% more phospho-GSK3β expression than controls, and PbA infected mice treated with LiCl had 203% higher expression of phospho-GSK3β. One-way ANOVA demonstrated a significant effect of treatment condition (F_(2, 18)_ = 54.82; p<0.0001) with post-hoc Tukey's test confirming significant different means between uninfected control and NaCl-treated ECM mice, and between uninfected controls and LiCl treated mice, but not between the two PbA groups. (**C**) There was no difference in the expression of total GSK3 between any of the groups. (**A, D–E**) There were no differences in phosphorylated tau or total tau protein expression between any of the groups. Phosphorylated tau levels returned to normal with CQ treatment with or without adjunctive lithium, and there were no group effects of treatment condition on protein expression of phosphorylated or total tau. For analysis, NaCl and LiCl treated control groups were combined as there were no significant differences in the means and no effect of treatment on mean protein expression. Total GSK3β and tau protein expression levels were normalized to GDI, and absolute total protein levels were used to normalize respective phosphorylated protein levels. Densitometry measurements are illustrated as a percentage of corresponding measurements in uninfected controls. Values plotted as mean ± SEM. n = 4 Na Con, 6 Na PbA, 6 Li PbA, 5 Li Con. **p<0.001. Na Con = NaCl treated control, Na PbA = NaCl treated *P. berghei* ANKA infected mice, Li PbA = LiCl treated *P. berghei* ANKA infected mice, Li Con = LiCl treated control. GDI was used as loading control.

There appeared to be a rebound inhibition of GSK3β in all ECM mice treated with CQ, with 220% more phospho-GSK3β expression in NaCl treated mice and 203% higher expression in LiCl treated mice when compared to uninfected control mice (Fig. 6B; p<0.0001). One-way ANOVA demonstrated a significant effect of treatment condition with post-hoc Tukey's test confirming significant different means between uninfected control and NaCl-treated ECM mice, and between uninfected controls and LiCl treated mice, but not between the two PbA groups. There was no difference in the expression of total GSK3 between any of the groups (Fig. 6C). Furthermore, there were no group effects on protein expression of phosphorylated or total tau between any of the groups (Fig. 6A, D–E). Phosphorylated tau levels returned to normal with CQ treatment.

**Figure 7:**
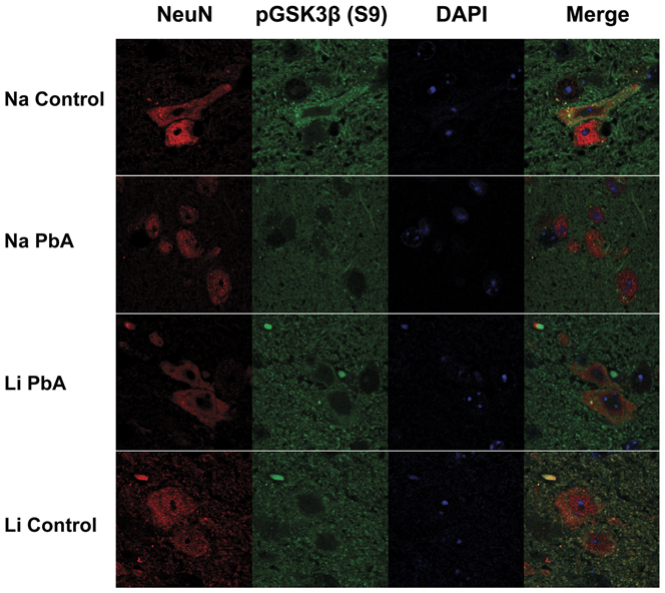
Immunofluorescence staining with NeuN (Red) and phospho-GSK3β (S9) (Green) in the brainstem. PbA-infected mice displayed different patterns in the distribution of phospho-GSK3β within neuronal cells (NeuN-positive), with a lack of phospho-GSK3β in neuronal nuclei in contrast to LiCl treated ECM mice and uninfected control mice treated either with or without LiCl. Na Con = NaCl treated control, Na PbA = NaCl treated *P. berghei* ANKA infected mice, Li PbA = LiCl treated *P. berghei* ANKA infected mice, Li Con = LiCl treated control, NeuN = neuronal nuclear antibody.

Interestingly, immunofluorescence analysis of brain sections (brainstem) demonstrated different patterns in the distribution of phospho-GSK3β within neuronal cells (NeuN-positive). Just as with acute ECM, PbA-infected mice treated with CQ without adjunctive LiCl exhibited a lack of phospho-GSK3β in neuronal nuclei in contrast to LiCl treated ECM mice and uninfected control mice (Fig. 7).

### Lithium effects on long-term cognitive impairment

**Figure 8:**
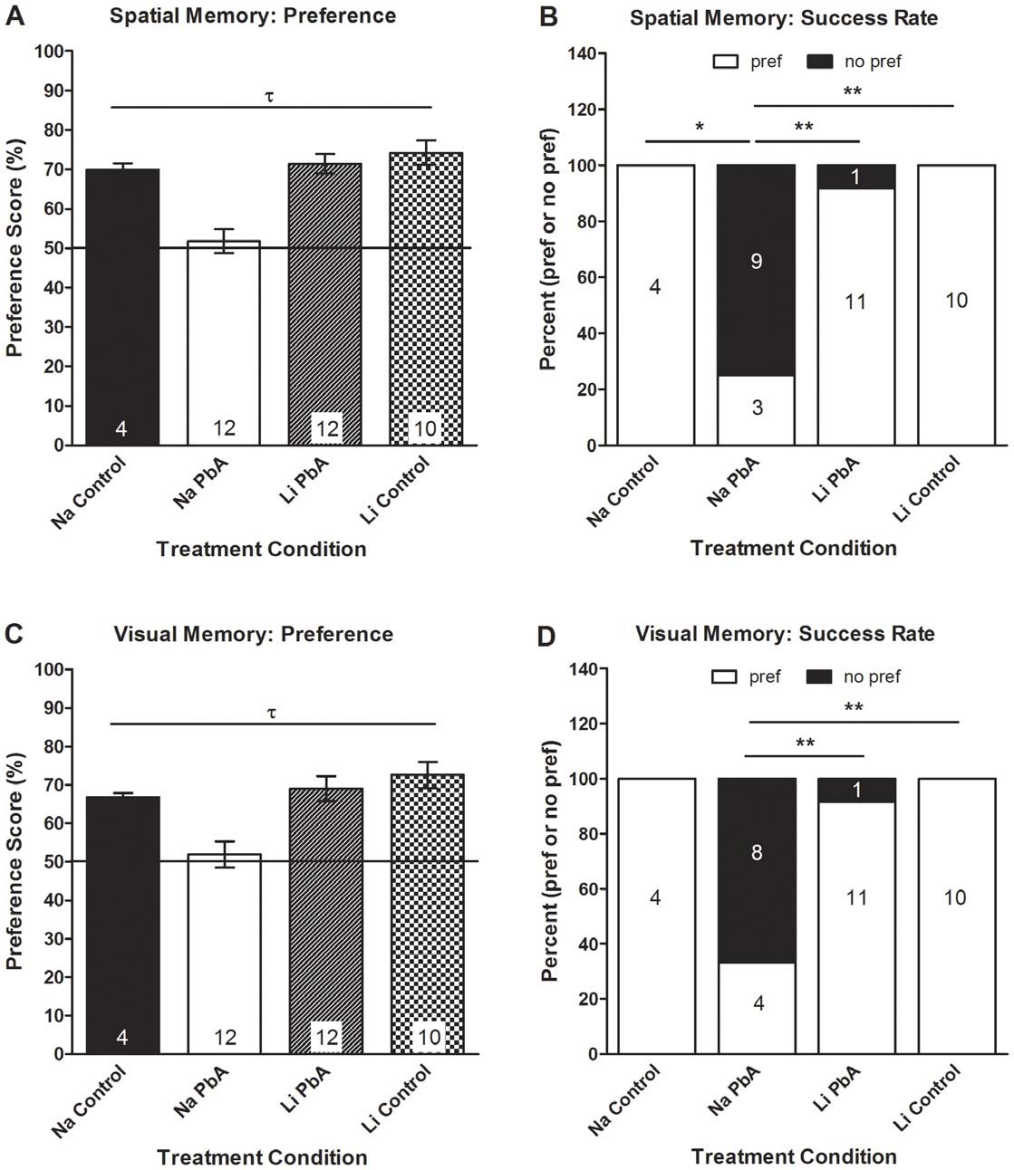
Effects of lithium on cognitive function in chloroquine treated mice. (**A**) There were significant group effects on mouse performance in the object placement test of spatial memory (F_(3, 34)_ = 13.56; p<0.001), with post-hoc Tukey's multiple comparison test confirming our previous observations [44] that PbA infection resulted in significantly lower preference scores than uninfected controls despite CQ treatment (Na PbA: 51.8±3.05 v. Na Con: 70±1.6 v. Li Con: 74.2±3.11). Adjunctive treatment with LiCl resulted in the prevention of spatial memory impairment as displayed in the object placement test, as PbA infected mice treated with LiCl scored significantly higher in the object placement test than NaCl treated PbA mice (Li PbA: 71.44±2.48 v. Na PbA: 51.8±3.05). (**B**) Moreover, a significantly higher proportion of NaCl-treated ECM mice showed spatial memory deficits (Na PbA: 75% v. Na Con: 0% v. Li Con: 0%), with a significantly lower proportion of LiCl-treated ECM mice exhibiting spatial memory deficits (Li PbA: 8.3% v. Na PbA: 75%). (**A–B**) There were no significant differences in the preference scores between the LiCl-treated ECM group and either uninfected control group, and no differences between sodium chloride and lithium chloride treated control mice. (**C**) Correspondingly, one-way ANOVA (F_(3, 34)_ = 7.951; p<0.001) demonstrated significant group effects on visual memory in the mice, with the lack of adjunctive lithium therapy in CQ-treated PbA mice resulting in significantly lower mean preference score in the object recognition test after a 45 min retention interval compared to uninfected controls (Na PbA: 51.92±3.42 v. Na Con: 69.8±1.1 v. Li Con: 72.59±3.41). Lithium treatment resulted in the prevention of visual memory impairment as ECM mice treated with LiCl had significantly higher preference scores in the object recognition test than mice receiving NaCl (Na PbA: 51.92±3.42 v. Li PbA: 69.01±3.28). (**D**) In addition, a significantly higher proportion of NaCl-treated ECM mice had deficits in visual memory compared to LiCl-treated ECM mice (Na PbA: 67% v. Li PbA: 8.3%) or uninfected, NaCl and LiCl treated control mice (Na PbA: 67% v. Na Con: 0% v. Li Con: 0%). (**C–D**) Just as with the object placement test, there were no differences in the preference scores of LiCl-treated ECM mice or in the proportion of LiCl ECM mice with a preference in the object recognition tests compared to either of the control groups. In addition, both sodium and lithium treated control mice performed similarly in the object recognition tests. n = 4 Na Con; 12 Na PbA; 12 Li PbA; 10 Li Con. *p<0.05, **p<0.01, τ = significant ANOVA F-test (p<0.05). Na Con = NaCl treated control, Na PbA = NaCl treated *P. berghei* ANKA infected mice, Li PbA = LiCl treated *P. berghei* ANKA infected mice, Li Con = LiCl treated control.

**Figure 9:**
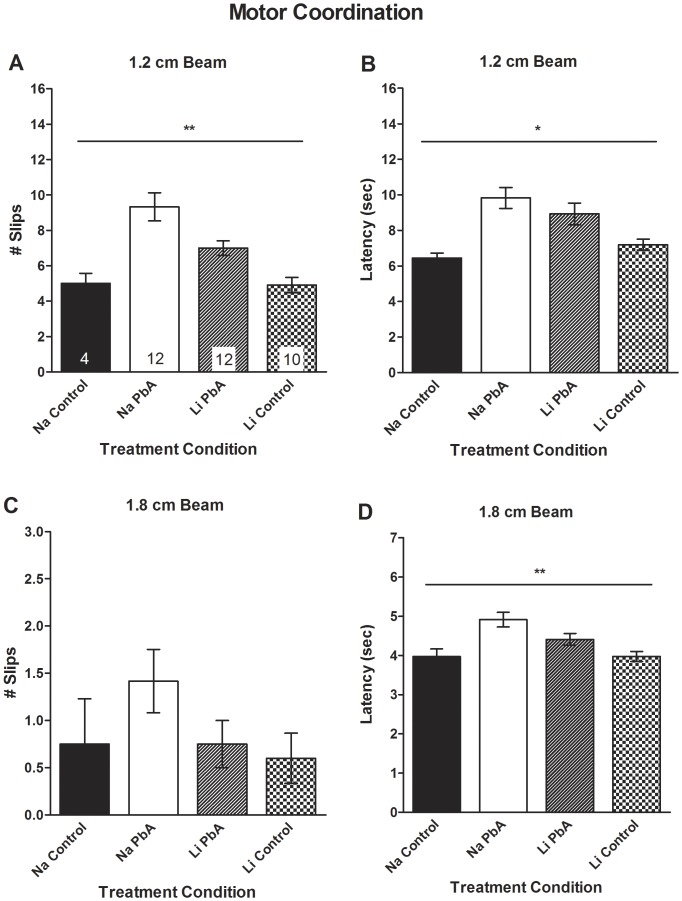
Effects of lithium treatment on motor coordination in chloroquine treated mice. (**A**) Consistent with our published observations [44], motor coordination deficits were evident in NaCl-treated ECM mice. A one-way ANOVA demonstrated significant group effects on the number of slips on the 1.2 cm beam (F_(3, 34)_ = 11.32; p<0.01), with PbA infection resulting in a significantly higher number of slips than uninfected control mice (Na PbA: 9.33±0.8 v. Na Con: 5±0.6 v. Li Con 4.9±0.4). LiCl treatment ameliorated the impairment in motor coordination in ECM mice as they experienced a significantly lower number of slips on the 1.2 cm diameter beam (Li PbA: 7±0.4 v. Na PbA: 9.33±0.8). LiCl-treated ECM mice still had significantly more slips than uninfected controls. (**B**) In addition, there were significant group effects on the latency to cross the 1.2 cm diameter beam (Na PbA 9.83±0.6 sec v. Na Con: 6.45±0.3 sec v. Li Con: 7.19±0.3 sec; F_(3, 34)_ = 6.27; p<0.01). Tukey's multiple comparison demonstrated that LiCl-treatment had no effect on the latency to cross the 1.2 cm beam when compared to NaCl-treatment in PbA-infected mice (Li PbA: 8.93±0.6 sec v. Na PbA 9.83±0.6 sec). (**C**) Although there were no significant differences among the four groups of mice in the number of in slips to cross the 1.8 cm diameter beam (F_(3, 34)_ = 1.56; p = NS), (**D**) there were significant effects of PbA infection on the latency to cross the 1.8 cm diameter beam compared to uninfected controls to cross the 1.8 cm diameter beam in ECM mice treated with NaCl (Na PbA: 4.91±0.2 v. Na Con: 3.97±0.2 v. Li Con: 3.97±0.1; F_(3, 34)_ = 6.85; p<0.01). Tukey's multiple comparison demonstrated that LiCl-treatment had no effect on the latency to cross the 1.8 cm beam when compared to NaCl-treatment in PbA-infected mice (Li PbA: 4.41±0.2 sec v. Na PbA: 4.91±0.2). (**A–D**) there were no differences in balance beam performance between LiCl-treated ECM mice and either of the control groups. In addition, both sodium and lithium treated control mice performed similarly in the motor coordination tests. n = 4 Na Con; 12 Na PbA; 12 Li PbA; 10 Li Con. *p<0.05, **p<0.001. Na Con = NaCl treated control, Na PbA = NaCl treated *P. berghei* ANKA infected mice, Li PbA = LiCl treated *P. berghei* ANKA infected mice, Li Con = LiCl treated control.

Long term treatment with lithium has been demonstrated to ameliorate performance deficits in transgenic mice expressing human amyloid precursor protein (APP) by inhibition of GSK3β [48]. In order to determine whether the lithium induced alterations in Akt/GSK3β signaling translated into favorable outcomes in CQ treated ECM mice, cognitive tests were performed cognitive and motor coordination tests on CQ-treated mice as per Dai et al [44]. Uninfected and PbA-infected mice were given either LiCl or NaCl from day 3 post infection (PI) until the cessation of a 10-day course of CQ treatment. Cognitive testing was performed 10 days after the cessation of CQ, and comparisons were made between each group. Consistent with our previous data [44], ECM mice with no adjunctive lithium treatment (NaCl-treated mice) had persistence of both spatial (Fig. 8A–B) and visual (Fig. 8C–D) memory and demonstrated motor coordination deficits after successful CQ treatment (Fig. 9). LiCl-treatment of ECM mice resulted in significant prevention of their cognitive and motor deficits.

There were significant group effects on spatial memory as assessed on the object placement test (Fig. 8A; p<0.001), with post-hoc Tukey's multiple comparison test confirming significant different means between NaCl treated ECM mice and both control groups and the LiCl treated ECM mice. Compared to both NaCl-treated and LiCl-treated control mice, NaCl-treated ECM mice demonstrated significantly lower preference scores in the object placement test of spatial memory (Fig. 8A). Moreover, a significantly higher proportion of NaCl-treated ECM mice showed spatial memory deficits (Fig. 8B; p<0.01). Compared to NaCl-treated ECM mice, LiCl-treated ECM mice demonstrated markedly higher preference scores (Fig. 8A), with a significantly lower proportion of LiCl-treated ECM mice exhibiting spatial memory deficits (Fig. 8B; p<0.01). There were no significant differences in the preference scores between the LiCl-treated ECM group and either uninfected control group (Fig. 8A–B), demonstrating that early lithium treatment prevents spatial memory deficits in ECM. There were no differences between sodium chloride and lithium chloride treated control mice (Fig. 8A–B).

Correspondingly, there were significant group effects, based on one-way ANOVA (Fig. 8C; p<0.001), in visual memory impairment in ECM mice as assessed by the object recognition test with post-hoc Tukey's test demonstrating significant effect of a lack of lithium treatment in PbA-infected mice on the mean preference score in the object recognition test. NaCl-treated ECM mice had significantly lower preference scores compared to NaCl-treated controls in the object recognition test of visual memory after a 45 min retention interval (Fig. 8C). In addition, a significantly higher proportion of NaCl-treated ECM mice had deficits in visual memory compared to LiCl-treated ECM mice (Fig. 8D; p<0.01) or uninfected, LiCl treated control mice (Fig. 8D; p<0.01). Just as with the object placement test, there were no differences in the preference scores of LiCl-treated ECM mice or in the proportion of LiCl ECM mice with a preference in the object recognition tests compared to either of the control groups (Fig. 8C–D). In addition, both sodium and lithium treated control mice performed similarly in the object recognition tests (Fig. 8C–D), illustrating that the addition of lithium to the anti-malarial regimen, early in the course of infection, prevents the long-term cognitive deficits incurred by mice with PbA infection.

### Lithium effects on long-term motor coordination impairment

Motor coordination was assessed on balance beams 10 days after the cessation of CQ in order to evaluate the effects of lithium on motor dysfunction. Lithium treatment resulted in a reduction of the long-term motor coordination impairment observed after successful CQ therapy in ECM mice.

Consistent with our published observations [44], motor coordination deficits were evident in NaCl-treated ECM mice, assessed as the number of slips (Fig. 9A; p<0.01) and latency to cross the 1.2 cm diameter beam, compared to both control groups (Fig. 9B; p<0.01). In addition, NaCl-treated infected mice took a significantly longer time than NaCl-treated controls to cross the 1.8 cm diameter beam (Fig. 9D; p<0.01). LiCl treatment partially prevented the impairment in motor coordination in ECM mice as they experienced a lower number of slips on the 1.2 cm diameter beam (Fig. 9A) and shorter latency to cross the 1.8 cm diameter beam (Fig. 9D) compared to NaCl-treated ECM mice. However, LiCl-treated ECM mice still had significantly more slips on the 1.2 cm diameter beam when compared to control mice (Fig. 9A) and similar latency as NaCl treated mice on both the 1.2 cm and 1.8 cm beams (Fig. 9B, D), indicating that lithium partially ameliorates the impairment of motor coordination in ECM mice. There were no significant differences between the four groups of mice in the number of in slips to cross the 1.8 cm diameter beam (Fig. 9C). In addition, there were no differences between NaCl and LiCl treated control mice (Fig. 9A–D). These data indicate that the effects of PbA infection on motor coordination were significantly ameliorated with adjunctive lithium treatment.

These data combined with the results of the cognitive tests suggest that lithium is neuroprotective and ameliorates or prevents the long term neurological deficits in our experimental model of cerebral malaria, and thus may be a potential agent for adjunctive therapy of cerebral malaria.

## Discussion

Neuronal metabolic dysfunction has been an under-recognized consequence of CM, and may play a role in the long-term neurological deficits that occur even when parasite burden is alleviated in CM. The phosphatidylinositol 3 kinase (PI3K)/Akt pathway is a cell survival pathway which is activated by insulin and several growth factors to affect a number of cellular functions including cell survival, apoptosis and glycogen synthesis. We demonstrate in this paper that there is a disruption in insulin signaling resulting in neuronal dysfunction upon infection with PbA in mice which is improved with adjunctive lithium therapy in chloroquine treated mice. Recently, Corby-Harris and colleagues demonstrated that regulation of the insulin signaling cascade is vital in *P. falciparum* development in mosquitoes. Activation of Akt signaling in the mosquito midgut not only significantly reduces parasite load in mosquitoes, but also reduces mosquito lifespan, effectively rendering them less infective to humans [41]. These studies imply that alterations in the effectors of the host PI3K/Akt signaling pathway are necessary for parasite growth, development and survival. Interestingly, our investigations demonstrate that alterations in Akt signaling carry over into mammalian hosts, where these changes can be detrimental to the mammalian host cells. In the setting of murine CM (ECM), we demonstrate that infection results in aberrant cerebral regulation of Akt/GSK3β signaling, a change that may result in abnormal cognitive function and long-term neurological sequelae. Interestingly, adjunctive lithium treatment, which was associated with a modest overexpression of phospho-Akt in infected mice, resulted in prevention of cognitive impairment and amelioration of motor-coordination dysfunction.

Previously, our laboratory group has demonstrated that ECM is associated with vasculopathy which results in a reduction of cerebral blood flow, neuronal impairment and cognitive deficits [43], [45], [49]. We demonstrated that ECM mice sustain memory deficits both during acute disease, in association with axonal damage and abnormalities in cells that maintain homeostasis of neurons, and after successful anti-parasitic treatment in surviving mice [43], [44]. With this study we demonstrate that ECM results in aberrant inhibition of the Akt cell survival pathway resulting in modifications of neuronal integrity. Akt, also called protein kinase B, is a serine/threonine kinase that is crucial for the insulin signaling pathway as well as an important regulator of cell survival and apoptosis. It promotes cell survival both by phosphorylating and suppressing the pro-apoptotic functions of certain caspases and by inhibiting the functions of forkhead transcription factors which regulate cell death genes, including the tumor suppressor p53. Akt promotes glycogen synthesis through phosphorylation and inactivation of GSK3β at Ser9. In addition, Akt is required for insulin-induced translocation of glucose transporter 4 (GLUT4) to the plasma membrane [39], [40], and is a critical effector of insulin and insulin-like growth factor (IGF-1)- mediated neuronal survival [38].

Upon activation with insulin or various growth and survival factors, such as the insulin-like growth factor (IGF-1), intrinsic tyrosine kinases on the cytoplasmic surface of the insulin receptors activate insulin receptor substrates IRS-1 and IRS-2 which then interact with the 85 kDa regulatory subunit of phosphatidylinositol 3- kinase (PI3K) to activate the 110 kDa catalytic subunit, setting off a cascade that results in the activation of Akt and the phosphorylation and inactivation of GSK3.

GSK3β is an important effector of Akt activity. GSK3β is a ubiquitously activated serine/threonine kinase, and a critical element of the PI3K/Akt cell survival pathway. It regulates glycogen storage by phosphorylating and inactivating glycogen synthase [50] and is essential in the regulation of processes such as cell metabolism, cell death and survival [33]–[37]. GSK3β has been implicitly linked to diverse medical problems, such as diabetes, cancer, neurodegeneration and Alzheimer's disease. Though GSK3β is ubiquitously expressed in all tissues, it is mainly found in developing and adult brains where it is most abundant in neurons [51].

GSK3β is important in the formation of neuronal polarity and dendrite extension during neuronal development [52]–[54]. In the adult brain, GSK3β plays a key role in the neuronal response to stress by phosphorylating and compromising the transcriptional activity of the cAMP response element binding (CREB), which regulates the transcription of the brain derived neurotrophic factor (BDNF) and other neuropeptides, important in the regulation of long-term memory, apoptosis and in maintenance of synaptic plasticity [55], thereby, contributing to the pathophysiology of neuronal degeneration [34], [56]. The enzyme has been demonstrated to be directly involved in the pathogenesis in neurodegenerative diseases such as Alzheimer's as a key kinase involved in tau regulation [32], [57], [58]. GSK3β is rendered inactive when it is phosphorylated at Ser9 by Akt [32].

We observed a decrease in the protein expression of Akt in our ECM model. This alteration decreases the inhibition of GSK3, leading to marked aberrations in the post-translational modification of tau protein. The aberrance in Akt/GSK3β signaling observed in PbA-infected mice may not only be etiologic in the abnormal neuronal profile detected in our model, including tau phosphorylation leading to conformational changes in the protein, but may contribute to the memory impairment demonstrated in these mice [43], [44].

Tau proteins are essential in assembly as well as maintenance of the structural integrity of microtubules [23]–[26]. When it is abnormally hyperphosphorylated, tau disengages from microtubules and increased cytosolic concentrations of unbound tau occur, resulting in neurofibrillary tangles [25]–[27], [59]–[64]. Hyperphosphorylated tau is the major component of the paired helical filaments that accumulate in degenerating neurons in Alzheimer's disease and other neurodegenerative diseases including fronto-temporal dementias [27]. These paired helical filaments are the principal features in the neurofibrillary tangles that are hallmark histopathological manifestations of Alzheimer's disease [25]–[27], [59]–[61], [65]–[67]. Several kinases including glycogen synthase kinase 3 (GSK3β), cyclin dependent kinase 5 (cdk5), and mitogen-activated protein kinase (MAPK) have been implicated in propelling tau hyperphosphorylation [25], [26], [66], [68]. Of these, GSK3β is probably the most documented kinase implicated in the abnormal hyperphosphorylation of tau [69]. Interestingly, abnormal tau in the cerebral spinal fluid has been shown to strongly correlate with disease severity and coma in both adult and pediatric cases of human CM [21], [22]. Our study corroborates these data and in addition, demonstrates that ECM results in aberrant phosphorylation of tau protein leading to conformational changes and accumulation of the unbound protein in neuronal cell bodies of PbA infected mice.

Based on our data, we believe that ECM causes alterations in the insulin signaling pathway and this affects neuronal function and survival. Neuronal damage as a result of metabolic dysfunction may explain the long-term deficits, including memory impairment, learning and language impairments, visuospatial and motor deficits, and psychiatric disorders, incurred by survivors of CM [1], [3], [6], [8], [70]–[74]. This mechanism likely leads to the development of neuronal damage in ECM.

A recent paper by Lacerda-Quieroz et. al. demonstrated a significant degree of inflammation and leukocyte recruitment to the cerebral microvasculature of C57BL/6 mice infected with PbN, yet these mice did not exhibit any abnormal neurological signs [75]. These observations support our theory that dysregulation in certain signaling pathways in neurons and other neural components such as Akt/GSK3β signaling greatly contributes to the adverse neuro-cognitive sequelae observed with ECM, and that these are not simply due to severe malarial disease or to the general inflammation or cerebrovascular damage usually observed with ECM. In this regard, increased Akt activation with lithium was associated with prevention of adverse neuro-cognitive outcomes suggesting a role for Akt in the long-term neurological impairment observed after CQ treatment in infected mice.

Lithium has recently been proposed to function as a neuroprotective agent which prevents neuronal apoptosis [76]–[79]. Though its mechanism of action remains unclear [76], lithium is known to both directly and indirectly regulate GSK3β through activation of the PI3K/Akt and MAPK signaling pathways in neurons [46], [47], [76] and by direct competition of mg2+ by binding to the catalytic site of the enzyme [80], [81]. This regulation of GSK3β is believed to be one of the main mechanisms by which lithium effects its neuroprotective role, as regulation of the kinase leads to downstream expression of anti-apoptotic and cell survival genes [76]. Lithium has been implicated in GSK3β-related reduction in glutamate excitotoxicity activity, suppression of p53 and increase in Bcl-2 expression involved in neuronal survival. Therefore, it is able to prevent brain damage following acute neural injury such as stroke [48], [76], and as demonstrated with these data, following cerebral malaria. Just as we demonstrated in our ECM model, long term treatment with lithium has been shown to ameliorate performance deficits in transgenic mice expressing human amyloid precursor protein (APP) by inhibiting GSK3β [48]. Here we demonstrate that both cognitive and motor coordination deficits were significantly ameliorated in PbA-infected mice who received early administration of lithium chloride in conjunction with CQ. This improvement in performance was associated with an increased Akt activation and an improved GSK3β phenotype in neurons. Although the lithium effect on GSK3 phosphorylation state in our model was no different than treatment with CQ alone, there was a qualitative difference in the distribution of phospho-GSK3β in neurons- particularly the restoration of intra-nuclear GSK3β expression.

CQ has been shown to have anti-inflammatory effects via inhibition TNF-α transcription and inhibition of the extracellular signal-regulated kinase (ERK) 1/2 and the mitogen-activated protein/ERK kinase (MEK) 1/2 [82], [83]. Thus, though there is no direct effect of CQ on Akt and GSK3 expression [84], the observed restoration of Akt and increased Akt activity- illustrated by the increased in phosphorylation of Akt substrates (i.e. GSK3β)- in the non-lithium treated ECM mice in our model may either be as a result of altered regulation of MAPK in ECM mice [85] induced by CQ treatment, or simply due to the general absence of inflammation after eradication of the parasite with CQ treatment [44]. Interestingly, inhibition of ERK has been shown to be associated with increased Akt response to IGF-1 in other models [86].

Incidentally, Nurul et al recently proposed that at high doses, lithium suppresses *P. berghei* parasitemia in mice [87] corroborating the findings by Corby-Harris and colleagues demonstrating that Akt overexpression in mosquitoes resulted in decreased *P. falciparum* load in the mosquito midgut [41]. Interestingly in our investigations, higher doses of lithium (50 mg/kg/day, or greater) was associated with adverse neuro-cognitive outcomes during treatment (data not presented); thus higher doses were not used in these investigations, and the anti-malarial properties of lithium in this model were not explored.

In this study, we used lysates from brain hemispheres, and this understandably limits our data as region-specific information would give us more direct information pertaining to the cognitive assays performed in this study. Future experiments involving the examination of specific brain regions will need to be performed for a more comprehensive assessment of the neuronal metabolic dysfunction observed in this paper. In addition, a time course will need to be performed in order to ascertain whether the protective properties of lithium will remain if adjunctive therapy is instituted at more advanced stages of disease, or once mice start to develop signs of ECM.

This report, to our knowledge, is the first to describe the dysregulation of Akt and GSK3β survival pathway and subsequent tau abnormalities as contributory to neuronal degeneration and to negative neuro-cognitive outcomes in ECM. Targeting this pathway by enhancing Akt activation with lithium administration in a ECM CQ-treatment model prevented the long-term abnormal cognitive phenotype associated with this model and ameliorated motor coordination in infected mice. We believe that this study will help shape the development of adjunctive therapy for prevention of neuronal damage with human CM.

## Materials and Methods

### Ethics Statement

This study was performed in strict accordance with the recommendations in the Guide for the Care and Use of Laboratory Animals of the National Institutes of Health. The experiments were approved by the Institutional Animal Care and Use Committee of the Albert Einstein College of Medicine. All efforts were made to minimize suffering.

### Animal infection and study design

6 week-old female C57BL/6 mice (Charles River Laboratories, Wilmington, MA) were infected via intraperitoneal (IP) injection with either 10^5^ red blood cells (RBC) parasitized with *Plasmodium berghei* ANKA (PbA) or 10^5^ RBCs parasitized with *P. berghei* NK65 (PbN) for comparison with a non-CM inducing malaria strain. Infected blood was diluted in phosphate-buffered saline (PBS) to provide the respective parasitized RBCs in a 200 µl injection. Uninfected age and sex-matched mice were used for comparison. Tail blood smears were stained with modified Giemsa stain (Sigma-Aldrich, St Louis, MO) and examined under a light microscope at days 4, 6 and 8 post-infection (PI). Mice were examined daily for locomotor activity and coat condition, and weight was measured every other day. Mice were euthanized using carbon dioxide, at day 8 PI for microscopic examination. Day 8 PI was chosen for assessment as it is the day when the majority of acutely PbA-infected mice are most symptomatic with low locomotor activity, raised fur, weight loss, prostration. These symptoms mimic the majority of cases of CM on presentation in endemic regions when major symptoms are evident. Cumulative data of survival studies over several experiments demonstrate a day 8 PI mortality of 75.5% in PbA infected mice and 21.1% in PbN infected mice when inoculated with 10^5^ infected red blood cells (iRBCs; see supplemental material, Fig. S1). Harvested brains were divided down the midline with half of the brain fixed in 10% normal buffered formalin and stored at room temperature, and the other half frozen in liquid nitrogen then stored at −80°C for protein extraction.

### Histology and Immunohistochemistry

The fixed brains were embedded in paraffin, sectioned at 4 µm thickness at the midline (sagittal sections) or at the following widths relative to bregma: 3.20, 0.38, −2.46, −4.04, and −6.48 mm (coronal sections). The sections were stained with hematoxylin-eosin. Two separate coronal sections were examined for each animal (n = 7 PbA; 4 Control; and 4 PbN).

The sections were deparaffinized and rehydrated, then boiled at 95°C for 20 min in sodium citrate solution (DAKO- Carpinteria, CA) for antigen retrieval. After a non-specific blocking step with hydrogen peroxide, the sections were incubated for 1 hour at room temperature with 5% (w/v) non-fat dry milk. For quantification of phospho-tau, the sections were incubated at 4° over 2 nights with PHF-1 [88], [89], a monoclonal antibody which recognizes tau protein phosphorylated at Ser396/404, at 1∶10 dilution, and MC-1 [89], [90] which recognizes a unique conformational epitope of misfolded tau protein, at 1∶50 dilution. A standard avidin-biotin complex method (Vector Immunolab, Burlingame, CA) was used for the secondary antibody (anti-mouse), using a kit from Vector Immunolab at 1∶200 dilution and a 2-h incubation period. Slides were counterstained with hematoxylin and dehydrated after immunolabeling. Photographs were taken using a Nikon Microphot-FXA microscope system and a Nikon digital sight DS-5M camera (Nikon Corporation, Japan).

For phospho-GSK3β (Ser9)/PHF-1 double staining, a polyclonal antibody to GSK3β (phospho S9) (Abcam, Cambridge, MA) was used in conjunction with PHF-1. Fluorescent labeled anti-mouse (Alexa fluor 594) and anti-rabbit (Alexa fluor 488) were used according to the manufacturers' instructions (Molecular Probes, Invitrogen, Carlsbad, CA). DAPI (Invitrogen) was used for counterstaining and ProLong® Gold antifade reagent (Invitrogen) was used as mounting medium. Images were processed on the Leica SP2 confocal microscope (Bannockburn, IL).

### Immunoblotting

Brain extracts were prepared in lysis buffer containing 50 mmol/l Tris pH 7.5, 1% NP-40, and 150 mmol/l sodium chloride plus protease inhibitor cocktail- and phosphatase cocktail (see protocol). Lysates were separated by sodium dodecyl sulfate (SDS) polyacrylamide gel electrophoresis and transferred to Supported Nitrocellulose Membrane (Bio-Rad Laboratories, Hercules, CA). Immunoblot analysis was performed using various antibodies as indicated. Monoclonal antibodies to PHF-1 and MC-1, monoclonal antibodies to total Akt and total GSK3β from Cell Signaling (Danvers, MA 01923), polyclonal antibodies to phospho-Akt (Ser473) and phospho-GSK3β (Ser9) from Abcam. The blotting membrane was incubated for 1 hour at 25°C in Tris buffer saline containing 0.1% (v/v) Tween20 (TBST) buffer supplemented with 5% non-fat dry milk (quantification of phospho-tau) or 5% BSA with 1% normal serum (quantification of total and phospho-Akt, GSK3β) to block nonspecific binding sites. After 1 hour incubation with primary antibody at 1∶500 dilution (PHF-1,MC-1, phospho-GSK3β) or at 1∶1000 dilution (total GSK3β, total and phospho-Akt) in TBST, the membrane was washed with the same buffer then blotted with the corresponding secondary antibody. Ponceau staining (Sigma-aldrich, St Louis, MO), and GDI (Invitrogen) were used as a loading controls. Primary and secondary antibodies were diluted in TBST and 5% BSA. Bound antibodies were detected by enhanced chemiluminescence according to the manufacturer's instructions (Amersham, Piscataway, NJ). Densitometry measurement was used to measure the intensity of the band specific to different antibodies. One-way analysis of variance were used using GraphPad Prism v5.04 (GraphPad Software, La Jolla, California) to compare the scores of control (n = 8), PbA (n = 7) and PbN (n = 6) infected mice. The experiments were replicated at least twice for PbN infected mice and greater than three times for PbA infected mice with similar results obtained in each trial.

### Lithium treatment and study design

Mice were randomly assigned into infected and control groups. Mice in the infected group were injected IP with 1×10^5^ RBC parasitized with PbA diluted in PBS to provide the respective parasitized RBCs in a 200 µl injection, and control mice were injected with uninfected blood diluted in PBS. Parasitemia was assessed by microscopic examination of tail blood smears stained with Giemsa (Sigma-Aldrich, St Louis, MO), every day from day 4 PI until the start of CQ treatment and every 3 days until the end of CQ to ensure complete parasite clearance. Baseline weight and blood glucose were recorded on the day of infection and monitored at day 2 PI, then every day from day 4 PI. Locomotor activity and coat condition were examined daily.

Each group of mice was then randomly assigned to daily treatment with either lithium chloride (LiCl) 20 mg/kg diluted in 200 µl of water or 0.9% sodium chloride (NaCl). LiCl/NaCl treatment was initiated at day 3 PI and was continued until the end of CQ therapy. This dose of lithium was chosen as it was the highest dose administered, over several dose/response trials, which did not confer immediate neurological side effects, an outcome measure in our experiments (data not presented). All mice, control and infected, were treated with a ten-day course CQ (20 mg/kg). CQ therapy was initiated when infected mice reached four pre-determined treatment criteria previously described [44], briefly, loss of >2% baseline body weight; glucose 20% lower than baseline; parasitemia >7%; and locomotor activity decrease of <50% control. CQ treatment was initiated at day 7 PI in control mice, the earliest day when infected mice first reached treatment criteria. Mortality was recorded and the experiments were replicated with a second cohort in order to obtain sufficient sample size and to determine the reproducibility of the results. Both cohorts underwent behavioral testing 10 days after cessation of CQ. Testing was performed with 4 NaCl control, 10 LiCl control, 12 NaCl PbA-infected and 12 LiCl control mice. Brains were harvested 7 days after the completion of testing for protein analysis with immunoblotting and immunohstochemical staining in order to both replicate data obtained from our previously published experiments [44] and to determine the effects of lithium treatment in chloroquine treated mice at that time point (n = 4 NaCl controls, 5 LiCl controls, 6 NaCl PbA, and 6 LiCl PbA mice).

### Cognitive tests and motor coordination assessment

As described previously elsewhere [44], to establish need for CQ treatment explained in the previous section, mice were allowed to move across a grid, and activity was assessed as the number of squares crossed and the number of rears, defined as lifting of the upper body and forepaws off the ground, in one minute. However, for locomotor assessment in the open field (post-CQ treatment), mice were placed in an opaque plastic arena (106 cm×106 cm square) for 9 min, and total track length was assessed with automated tracking software (Viewer: Biobserve, Bonn, Germany).

Behavior tests were performed as previously described [44]. Animals in each of the four treatment groups received 2 trials – a familiarization or sample trial (Trial 1) and a test trial (Trial 2). In the object recognition test, mice were placed in an open field and allowed to freely explore two identical objects for 3 minutes then returned to their home cage (Trial 1). Object exploration was recorded manually with a timer, and was defined as physical search of the object including touching, whisking, sniffing, and rearing toward the objects or facing to the objects within the distance of 2 cm. After a retention interval of 45 minutes in their home cage, mice were replaced in the open field with the one of the familiar objects and a novel object, and they were allowed to freely explore for 3 minutes (Trial 2). The object placement test was conducted similarly except that animals were allowed to explore for 5 minutes in trial 1 and in trial 2 the same objects were used, but one was displaced in space. Retention intervals in the object placement task were 30 min.

In both assays, normal animals preferentially explore the novel (or displaced) object. Data are presented as a preference score (time spent exploring novel object/total time spent exploring both objects×100). A preference score of 50% indicates chance performance. Data are also presented as the “success rate.” or the proportion of animals with a novel object preference of 55% or better in each experimental group. The experimenter was blind to the condition of the animals. All objects and positions were counterbalanced and all pairs of objects have been previously validated.

As previously described [44], balance beam tests were performed. Mice first received 3–4 pretraining trials on a plank of 4 cm wide. Mice were then tested for motor coordination on 2 balance beams (65 cm long) of varying difficulty – a thinner beam, 1.2 cm diameter, and an easier beam, 1.8 cm diameter. Motor coordination was assessed as the number of slips and latency to cross the beam. Grip strength was assessed by latency to fall when suspended by the forepaws from a wire.

### Statistical analysis

All statistical analysis compared the infected groups to the control group, or compared the treatment groups to the control groups with the level of significance at p<0.05, p<0.01 and p<0.001. Parasitemias at different time points were assessed by T-tests. Hemorrhage scores, Immunoblot densitometry scores, preference scores, number of slips, latencies and other normally distributed data were statistically analyzed by One-way ANOVA with post-hoc Tukey's multiples comparison analysis using GraphPad Prism. Success rate in the cognitive tests were analyzed by Chi Square tests (JMP: SAS, Cary. NC).

## Supporting Information

Figure S1 **Progression of parasitemia and survival with chloroquine (CQ) and lithium treatment.** (**A**) PbA infected mice randomly assigned to daily treatment with either lithium chloride (LiCl) 20 mg/kg or 0.9% sodium chloride (NaCl), initiated at day 3 PI, were treated with CQ (20 mg/kg). Infected mice exhibited similar parasitemia levels prior to the insitution of CQ therapy with both groups peaking at day 7 (Na PbA: 8.5%±0.68; Li PbA: 9.4%±0.82; p = NS). (**B**) There was no effect of LiCl treatment on survival (54% both groups). All mice received CQ treatment. n = 24 Na PbA; 24 Li PbA; 5 Na Control; 10 Li Control. Na Control = uninfected control mice treated with sodium chloride (NaCl), Li Control = uninfected control mice treated with lithium chloride (LiCl), Na PbA = *P. berghei* ANKA infected mice treated with NaCl, Li PbA = *P. berghei* ANKA infected mice treated with LiCl.
(TIF)

Figure S2 **Cumulative illustration of progression of parasitemia and survival during PbA and PbN infection.** (**A**) There is a gradual increase in the percent parasitemia in both PbA and PbN infected mice. On the day of brain harvest (day 8 post-infection (PI)), the parasitemia is usually 14.8%±6.14 in PbA infected mice and 6.3%±0.96 in PbN infection. (**B**) Survival is usually 24.5% in PbA-infected mice and 78.9% in PbN-infected mice at day 8 PI. n>40 PbA, >30 PbN. PbA = *P. berghei* ANKA infected mice, PbN = *P. berghei* NK65 infected mice.
(TIF)
