# Supplementary figures and images for: Altered Regulation of Akt Signaling with Murine Cerebral Malaria, Effects on Long-Term Neuro-Cognitive Function, Restoration with Lithium Treatment
Source: PLoS One. 2012 Oct 17;7(10):e44117. doi: 10.1371/journal.pone.0044117 (PMC3474787; doi:10.1371/journal.pone.0044117)

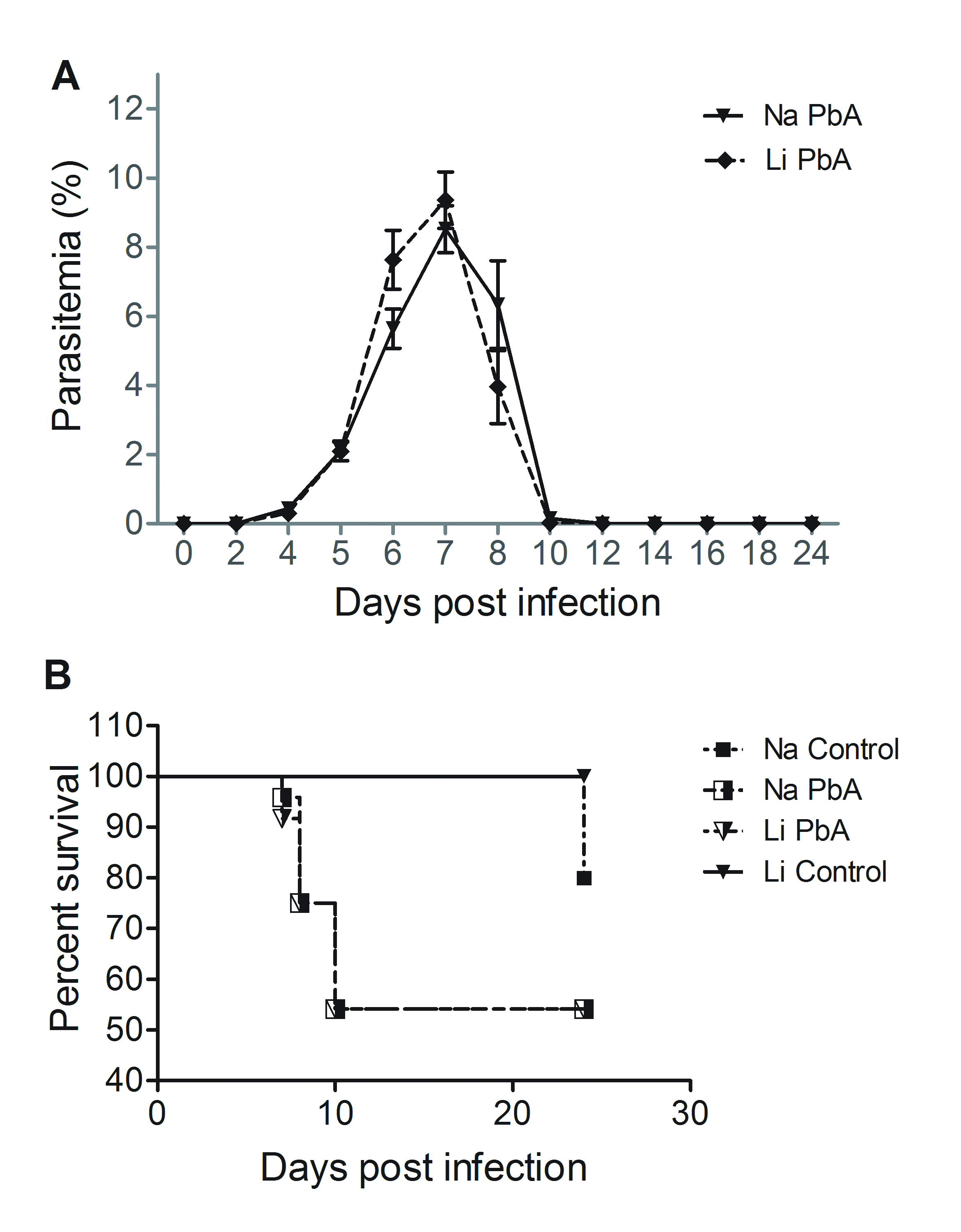

Supplement: Figure S1 — Progression of parasitemia and survival with chloroquine (CQ) and lithium treatment. (A) PbA infected mice randomly assigned to daily treatment with either lithium chloride (LiCl) 20 mg/kg or 0.9% sodium chloride (NaCl), initiated at day 3 PI, were treated with CQ (20 mg/kg). Infected mice exhibited similar parasitemia levels prior to the insitution of CQ therapy with both groups peaking at day 7 (Na PbA: 8.5%±0.68; Li PbA: 9.4%±0.82; p = NS). (B) There was no effect of LiCl treatment on survival (54% both groups). All mice received CQ treatment. n = 24 Na PbA; 24 Li PbA; 5 Na Control; 10 Li Control. Na Control = uninfected control mice treated with sodium chloride (NaCl), Li Control = uninfected control mice treated with lithium chloride (LiCl), Na PbA = P. berghei ANKA infected mice treated with NaCl, Li PbA = P. berghei ANKA infected mice treated with LiCl. (TIF) [file pone.0044117.s001.tif]

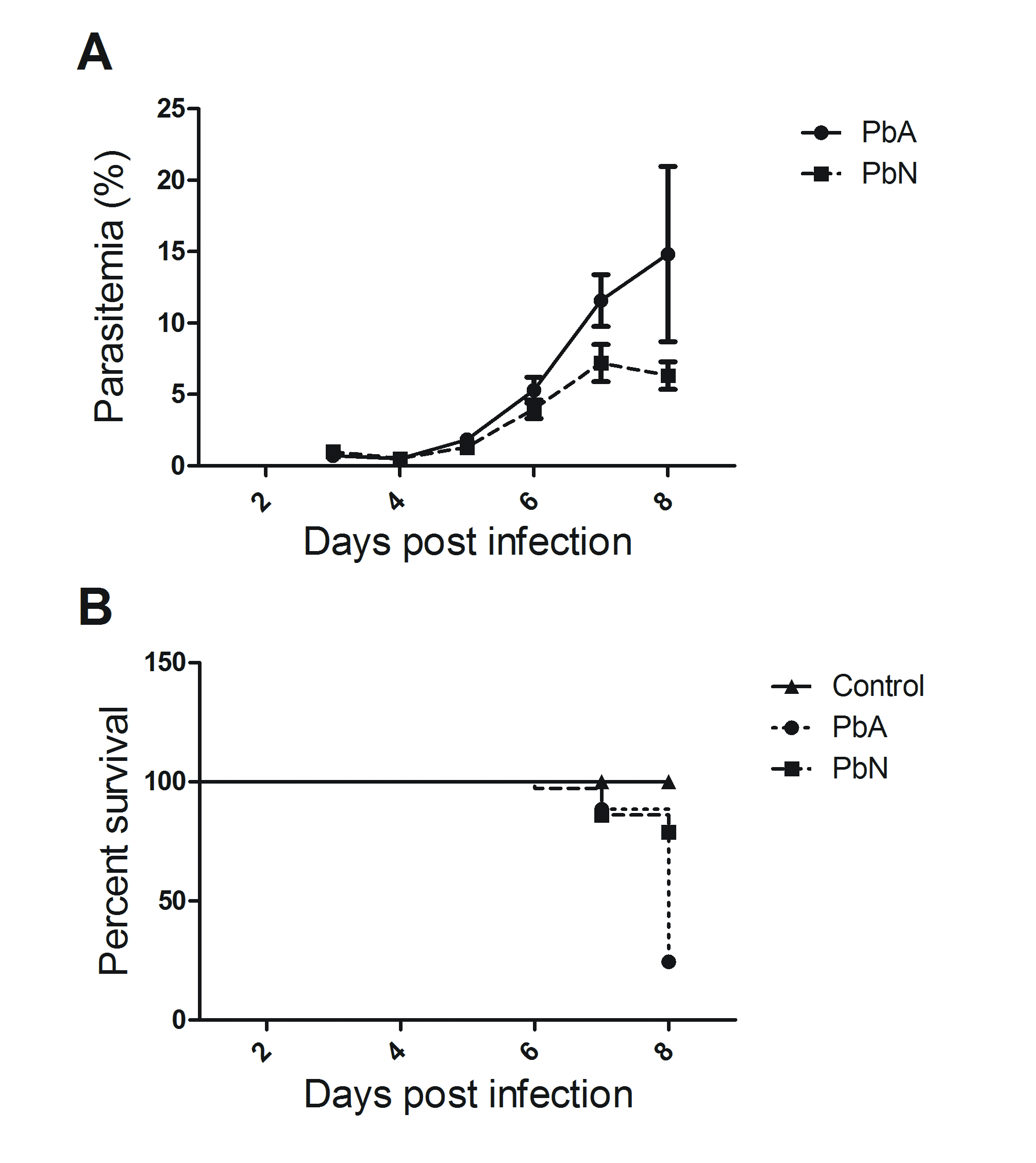

Supplement: Figure S2 — Cumulative illustration of progression of parasitemia and survival during PbA and PbN infection. (A) There is a gradual increase in the percent parasitemia in both PbA and PbN infected mice. On the day of brain harvest (day 8 post-infection (PI)), the parasitemia is usually 14.8%±6.14 in PbA infected mice and 6.3%±0.96 in PbN infection. (B) Survival is usually 24.5% in PbA-infected mice and 78.9% in PbN-infected mice at day 8 PI. n>40 PbA, >30 PbN. PbA = P. berghei ANKA infected mice, PbN = P. berghei NK65 infected mice. (TIF) [file pone.0044117.s002.tif]
